# Couples-based interventions in the context of HIV discordance

**DOI:** 10.4102/sajpsychiatry.v23i0.1009

**Published:** 2017-05-30

**Authors:** Sibongile Mashaphu, Jonathan K. Burns

**Affiliations:** 1 Nelson R Mandela School of Medicine, Department of Psychiatry, University of KwaZulu-Natal

Until very recently, a diagnosis of HIV was associated with social stigma and chronic debilitation, and imminent death was the most likely outcome. It is well established that persons with HIV infection have high rates of stressful life events because living with HIV presents multiple challenges that often overwhelm coping resources and impair psychological adjustment to the ongoing demands of managing this highly stigmatised, chronic illness.

Research indicates that psychological interventions may impact favourably on immune status in HIV-positive persons and improve general well-being.^1^ Positive psychological states (such as positive effect) promote resilience in the face of negative life events and facilitate more effective management of HIV infection. This in turn independently predicts less rapid HIV disease progression.

In recent times, with improving health care and increased ARV uptake, the life expectancy and overall quality of life for those living with HIV have improved substantially. The successes of VCT campaigns are also beginning to yield good results, with more people electing to take control of their lives by voluntarily testing to know their HIV status and striving to remain uninfected. With such effective treatment advances, long-term HIV survivors are turning their attention back towards careers and relationships, electing to live a healthy, meaningful life with HIV as one of many chronic illnesses that afflict our communities.

Invariably, at some point, some individuals not infected with HIV may make a conscious decision to enter into a relationship with someone already living with HIV. This type of partnership is referred to as a mixed HIV status or sero-discordant couple. As health care providers, we should not frown upon such individuals or couples, but rather provide them with the necessary tools to navigate this potentially volatile partnership by creating appropriate and relevant counselling programmes.

Importantly, the prevalence of sero-discordant relationships is growing in South Africa for a variety of reasons. Discordance ranges from 20% in the general population to 51% within couples in which one partner seeks HIV care.^2^ Of great concern is the fact that HIV is most frequently transmitted within the context of partners in a committed relationship, raising important issues such as risk of infection, reproductive choices and stress on relationships themselves.

The issue of HIV sero-discordance is difficult for most to conceptualise and is poorly understood by most couples as well as most health care providers tasked with looking after them. The question of why certain individuals do not readily seroconvert to a positive status despite multiple exposures to the virus remains a huge puzzle for scientists, especially because most new HIV infections in Africa now occur in cohabiting couples, many of whom do not realise that only one may be infected because of either non-disclosure or infidelity.

From the beginning of the HIV epidemic, relationships comprising one positive and one negative partner have been fraught with problems leading to one or both partners seeking counselling or even psychiatric care. Sources of conflict usually centre on how the illness was acquired, fear of transmission, concerns with procreation and guilt. Regrettably, in some relationships, domestic and gender-based violence surfaces once the positive status of one partner has been disclosed or acknowledged. Feelings of anger, hopelessness, symptoms of depression, anxiety and, in some cases, suicidal ideation may arise.^1^

In general terms, the behavioural aspects of HIV infection are incredibly complex and require close inspection and analysis in order to provide relevant and appropriate interventions.^1^ Notably, HIV prevention interventions generally focus on individuals at risk, rather than specifying couples as the unit of change. Such interventions often neglect the crucial role that partners play in sexual behaviour. A closer look at the VCT literature reveals that most individuals report having received counselling about their HIV status, but with little or no emphasis on their relationships. Although in South Africa (as in many countries) the preference is for provider-initiated testing and counselling, and many health care providers and counsellors have been trained to provide basic counselling, the majority still lack specific training to address the complex needs of couples living with HIV.^3^

Moving beyond the context of HIV testing and counselling, couples-focused interventions may differ from individual-focused HIV prevention programmes by addressing the ongoing dynamics that contribute to sexual risk behaviour, power imbalances, commitment, satisfaction and intimacy.^1^ Interventions to reduce transmission between sexual partners are undoubtedly an essential component of efforts to curtail the epidemic; psychological interventions designed to improve psychological adjustment (i.e. decrease negative effect as well as enhance positive psychological states) may encourage behaviour change, modulate stress physiology and slow HIV progression.

Although couples-based HIV prevention interventions have been promoted as a potentially promising strategy, synthesising the effectiveness of these programmes is challenging because methods vary.^3^ For example, it is not clear whether the target of interventions should be the individual, the couple or even a group of couples. Furthermore, as evident from the Couples HIV Testing and Counselling (CHTC) in Health Facilities programme developed by the Centers for Disease Control and Prevention (CDC) and implemented in more than 22 countries including South Africa, there are many practical challenges with implementation of couples-based interventions.^3^ These include providers saying they are too busy to conduct CHTC, couples not coming together for counselling and testing, and providers fearing conflict between couples when discordance is disclosed.

Although such challenges are real, it is our responsibility as health practitioners to overcome these barriers in order to make an impact on the HIV epidemic. In the author’s view, HIV counselling and testing facilities and independent practitioners in South Africa should be supported with the capacity to address issues related to depression, anxiety, sexual violence and many other unique needs for couples who are HIV discordant. Recognising the unique challenges of these couples as well as their emotional, psychological and physical needs should form an integral part of our holistic global and national responses to the HIV epidemic.

Further research on the reproductive, psychological and social needs of discordant couples may also be beneficial in the global response to HIV, informing further appropriate interventions. The recent successes of targeted-population programmes such as prevention of mother-to-child transmission, rape survivors (PEP) and, recently, male medical circumcision give us confidence that a couples-based intervention, guided by evidence and tailor-made for the South African public, would also be successful and contribute positively to curtailing the epidemic and improving the quality of life of those living with HIV.
